# Attitudes of Doctors and Nurses toward Patient Safety within Emergency Departments of a Saudi Arabian Hospital: A Qualitative Study

**DOI:** 10.3390/healthcare7010044

**Published:** 2019-03-18

**Authors:** Naif Alzahrani, Russell Jones, Mohamed E. Abdel-Latif

**Affiliations:** 1 The Medical School, College of Health and Medicine, Australian National University, Acton, ACT 2601, Australia; 2 Emergency Services Research Group, Health Simulation Centre, School of Medical and Health Sciences, Edith Cowan University; Joondalup, WA 6027, Australia; russell.jones@ecu.edu.au; 3 Department of Neonatology, Centenary Hospital for Women and Children, Canberra Hospital, Garran, ACT 2605, Australia; Abdel-Latif.Mohamed@act.gov.au

**Keywords:** patient safety climate attitudes, hospital emergency department, qualitative

## Abstract

*Background:* The attitudes of doctors and nurses toward patient safety representa significant contributing factor to hospital safety climates and medical error rates. Yet, there are very few studies of patient safety attitudes in Saudi hospitals and none conducted in hospital emergency departments. *Aims*: The current study aims to investigate and compare the patient safety attitudes of doctors and nurses in a Saudi hospital emergency department. *Materials and Method:* The study employed a qualitative research design via semi-structured interviews with Saudi and non-Saudi doctors and nurses working in a Saudi hospital emergency department to determine their attitudes and experiences about the patient safety climate. *Results:* Findings revealed doctors and nurses held some similar safety attitudes; however, nurses reported issues with doctors with respect to their teamwork, communication, and patient safety attitudes. Moreover, several barriers to the patient safety climate were identified, including limits to resources, teamwork, communication, and incident reporting. *Conclusion*: The findings provide one of the few research contributions to knowledge regarding the patient safety attitudes of Saudi and non-Saudi doctors and nurses and suggest the application of such knowledge would enhance positive patient outcomes in emergency departments.

## 1. Introduction

The attitudes of doctors and nurses toward patient safety representa significant factor in hospital safety climates and medical error rates [1]. “Safety climate” can be defined as the perceptions about how safety is managed within an organisation in terms of measurable components such as management behaviours, safety systems, and employee safety attitudes [2]. Indeed, more positive safety attitudes among nurses and doctors have been found to be associated with lower medical error rates [3]. Given the high rate of patient medical errors and related costs [4], knowledge about how to improve the patient safety climate attitudes in hospital settings has the potential to improve clinical outcomes and contribute to organisational efficiencies.

Quantitative survey research on patient safety climate attitudes has been conducted in a range of countries and hospital departments, showing doctors and nurses generally report more positive attitudes towards the dimensions of hospital safety climate and teamwork and less positive attitudes on the dimensions ofhospital working conditions and the quality of management support [5,6,7]. Research in the context of Saudi Arabian hospitals has similarly shown less positive attitudes among nurses and doctors with respect to management support and working conditions [8,9]. Nevertheless, few studies have investigated patient safety climate attitudes of hospital staff in-depth via qualitative methodologies.

The current study aims to investigate the patient safety climate attitudes of doctors and nurses employed in the emergency department of a Saudi hospital by employing a qualitative research design. Qualitative research provides the advantage of being able to probe the internal states, beliefs, and attitudes of participants [10]. At the same time, the research in this study addresses a gap in the literature as there have been very few studies of patient safety climate attitudes in Saudi hospitals and none conducted in hospital emergency departments.

## 2. Materials and Method

### 2.1. Study Design

The study employed a qualitative research design by collecting data via semi-structured interviews with Saudi and non-Saudi doctors and nurses working in a Saudi hospital emergency department to determine their attitudes and experiences about the patient safety climate. A qualitative design with semi-structured interviews was considered a suitable method to develop a richer and deeper analysis of patient safety attitudes [11]. The data from interviews weresubjected to thematic assessment via Interpretive Phenomenological Analysis (IPA) [11,12] to identify the significant meanings behind participant’s patient safety climate attitudes.

### 2.2. Participants

The participant inclusion criteria were doctors and nurses who worked in the emergency department of a Saudi hospital; all other hospital staff were excluded from participation in the study Ten nurses and 10 doctors participated in interviews, which was considered to be an appropriate number for this type of research given data collection reached saturation point [13], and due to the resource constraints of qualitative research (time and opportunity). The final sample of participants included 5 Saudi doctors, 5 non-Saudi doctors, 3 Saudi nurses, and 7 non-Saudi nurses.To protect their identity, each participant was assigned a code: SD1 to SD5 (Saudi doctors); NSD1 to NSD5 (non-Saudi doctors); SN1 to SN3 (Saudi nurses); and NSN1 to NSN7 (non-Saudi nurses). More specific details about the professional background of participants werenot collected due to concerns about privacy and confidentiality.

### 2.3. Setting

The study was conducted in the emergency department of a Ministry of Health (MOH) hospital in Riyadh, Kingdom of Saudi Arabia. This hospital receives more emergency cases than any other hospital in the kingdom. The hospital has 1400-bed capacity with more than 8000 employees [14]. The emergency department receives approximately 160,000–180,000 patients annually [14].

### 2.4. Data Collection

Data collection entailed semi-structured interviews with doctors and nurses about their experience of safety attitudes and climate in the emergency department. Based on a review of the patient safety climate research literature and consistent with the recommendations of Spradley [15], the interview questions followed four general types consistent with the framework of IPA. These were: descriptive (e.g., What are the important facilitators and barriers to patient safety in your department?), structural (e.g., In your perspective what are the important elements of a successful emergency department safety climate?), contrast (e.g., How do problems in factors like teamwork, management support and job setting translate into patient safety issues?), and evaluative (e.g., What is your evaluation of the safety attitudes in your department?). The duration of interviews was between 15 min and 30 min and interviews were recorded, transcribed, and transferred to the data analysis package NVivo 11 for qualitative analysis and thematic coding [16]. A full list of interview questions is shown at Annex 1.

The datasets used and/or analysed during the current study are not publicly available as individual participant confidentiality could be jeopardized, and participant confidentiality was required for approval of the study by the participating bodies. Aggregate summaries of the data are, however, available from the corresponding author upon reasonable request and with permission of the participating hospitals. All authors had full access to all the data (including statistical reports and tables) in the study and can take responsibility for the integrity of the data and the accuracy of the data analysis.

### 2.5. Data Analysis

Data analysis commenced with the process of immersion [17] which entailed listening to the recording of each interview after its conclusion in order to review the content and record any observations in field notes [12]. The next step focused on the coding of participant responses to each interview question to note patterns and common themes that appeared in the data. Using this approach, codes were assigned to represent and categorize the main patterns of meanings in the interviews [16,18]. By this process, the codes were further defined, described, and linked together into groups to produce a list of main themes and subthemes of meaning associated with patient safety attitudes.

A range of steps were taken to establish the credibility, dependability confirmability, and transferability of the data [19]. To establish the credibility of the findings, descriptive field notes were taken during interviews to document observations and added context to the audio data. Confirmability of findings entailed the use of analytic memos to ensure objectivity in any interpretations made during the course of data analysis [20]. The transferability of research findings was met by purposive sampling of participants based on their capacity to provide relevant knowledge on emergency department safety climate attitudes [20]. The criteria of ensuring dependability was met by having an outside researcher conduct an inquiry audit on the research study.

### 2.6. Ethical Consideration

The investigation was aligned with important principles associated with research with humans. These principles included respect for justice, autonomy, beneficence or do good, and nonmaleficence or do no harm [21]. 

Ethics approval and consent to participate: The study was approved by Australian Capital Territory Health Research Ethics Committee (ETHLR.16.247); Australian National University Human Ethics Committee (Protocol 2017/514) and the General Directorate for Researches and Studies, Ministry of Health, Kingdom of Saudi Arabia. The participants received oral and written information about the study, including details about confidentiality in handling the data. The participants were also informed about the voluntary nature of their participation, including the fact that they could terminate their participation at any time. Written informed consent was obtained from each participant prior to their participation.

## 3. Results

Five main themes were derived from the coding of the interview data on patient safety climate attitudes. These were safety evaluations, safety barriers, safety facilitation, safety issues, and safety needs. Moreover, four of these themes were comprised of a series of subthemes to represent specific safety climate attitudes, including a total of 12 subthemes altogether. A full list of the themes and subthemes is reported in Table 1. This section provides an in-depth presentation of the findings with respect to each main theme and sub-themes through reference to the respondent’s own words.

### 3.1. Safety Evaluation Theme

Whilefour out of five of the Saudi doctors had a favourable evaluation of the safety climate, all the non-Saudi doctors reported a less than positive evaluation of the safety climate. Similarly, most Saudi and non-Saudi nurses reported a less than positive rating of the safety climate in the emergency department. The findings also showed some nurses had unfavourable ratings of doctors’ safety climate attitudes: “*For nurses, they are good. They are doing their best to improve patient safety and they are trying to do their best. For doctors it depends some physician, they are doing their best, but others, no. They don’t care*” (SN2).

### 3.2. Barriers to Patient Safety Theme

Participants reported their perceptions of a range of barriers to the patient safety climate which reflected three subthemes: accountability and blame, medical errors, and language. The most frequently mentioned barrier to safety was language, which reflects the multi-cultural nature of Saudi society. In fact, 15 of the 20 participants reported that language barriers couldbe a problem when communicating to patients. This is exemplified by a non-Saudi nurse who stated: “*The patient, they are saying something, we cannot understand. We’re not fluent in Arabic, and sometimes the patient is saying some important information that we need to know in order to give them the proper care*” (NSN6). In contrast, participants did not view religion, national differences, or gender to be significant barriers to patient safety in the emergency department: “*There are no differences, as long as you’re working in the hospital the patient safety should come first*” (NSN4).

In terms of the Safety barriers subtheme of Medical Errors, participants mostly reported patient safety issues with infection control and prescribing medications. For example, one Saudi doctor shared: “*There are some scattered problems like infection control and this could affect patient safety*” (SD2). A small number of participants also reported two other important barriers to safety climate in the subthemes of accountability and blame. Indicating that a lack of accountability can impact negatively on patient safety climate, one Saudi nurse shared: “*The most important barrier for me is the accountability. If you have incident, and you report it one and two and three times, there is no accountability to make people more compliant with the patient safety*” (SN2). Three participants also highlighted that attributing blame for incidents can act as a barrier to patient safety climate. As put by one non-Saudi nurse: “*I think the most important factor in order for us to have safety for our patients is having a non-blame environment, because if, for example, when the staff commit a mistake, we found out there is an error of medication or she did something wrong, and then we will punish this staff. The next time around, the other staff will not report it anymore*” (NSN1).

### 3.3. Safety Facilitation Theme

Staff teamwork was the most frequently mentioned safety climate facilitation subtheme. Participants generally held positive attitudes towards the teamwork of fellow staff and this was similar between Saudi and non-Saudi doctors and nurses. Importantly, participants also reported the link between teamwork and optimal patient outcomes*:* “*We have a good teamwork here, and we have more support from the management. Translating (evidence) into patient safety (is also supported)*” (NSN4). Nevertheless, there was mention by non-Saudi nurses of problematic teamwork associated with the attitudes of doctors: “*Most of the time we have good team. But there are some times that the doctors have maybe some problems with the attitudes. So, they could be rude sometimes or shout out to you. So, if you have any concern, you couldn’t ask them*” (NSN7).

In contrast to the generally positive perceptions of staff teamwork, participants reported mixed views about management support to facilitating the patient safety climate. Whereas participants reported modest and improved support, there was the view that more could be done to support frontline staff to develop the patient safety climate. As one Saudi doctor stated: “*Management support, it is very important to support your staff as this will improve the quality and patient safety*” (SD2).

Like the perceptions of management, attitudes towards the safety facilitation subtheme of communication were less than positive. Most nurses had less than positive attitudes about their communication with doctors. At the same time, doctor’s communication with patients was also said to be lacking by one non-Saudi nurse, who made the link to the effects of poor communication on patient safety by stating: “*There are still some doctors who will not explain the diagnostic procedure that they are undergoing with the patient. That’s the time now there is a break with the safety of the patient*” (NSN1).

A final subtheme to be derived from the data was how participants employed protocols and incident reporting to facilitate patient safety. Although participants indicated that there are protocols to follow for reporting patient safety incidents, such as the Occurrence Variance Report (OVR), there was a general view among doctors and nurses that staff were under-reporting. This was seen to be due to the perception that there was little or no follow up to incident reporting or the report being perceived as a complaint: “*Actually, at the beginning, we were reporting, but with no feedback, so, for me I stopped reporting*” (SD4).

### 3.4. Safety Issues Theme

The most frequently reported safety issue was problems with facilities, resources and equipment. One non-Saudi nurse elaborated on the negative impact of unavailable resources in terms of the challenge it presents to patient safety: “*This started when they cut off the supply from Ministry of Health. So, if we are lacking resources, the staff will not do things right. For example, if the cleaning material is not available, they will not clean anymore. If the alcohol swab is not available, they will not clean anymore, because they know it’s limited. They want it to be resourceful enough to make ends meet in order for the supply to be available for 24 h*” (NSN1).

A further subtheme of safety issues identified in the data was the view that some staff do not comply with safety standards which may have negative implications for patients and staff alike. A Saudi doctor spoke to the issue of uncompliant staff by stating: “*Sometimes our staff compromise themselves to high risk patient. They knew that this patient has a high risk of respiratory infection symptoms and they need to wear special mask such as N95 mask, but they are not using this*” (SD5). In terms of doctor compliance to provide patient safety, one Saudi nurse observed: “*For doctors it depends. Some physicians, they are doing their best, but others, no. They don’t care[about patient safety]*” (SN2). A further patient safety issue subtheme was the problem of overcrowding and work overload reflecting poor staff-patient ratios. As one Saudi doctor shared: “*The overload, overcrowded, multiple admitted patients, this affects patient safety directly*” (SD5). Moreover, this safety issue was further elaborated by a non-Saudi nurse who stated: “*Considering the fact of the number of patients coming and the workload that the staff is receiving, the staff could be prone to errors, and I couldn’t say it’s that safe*” (NSN7).

### 3.5. Safety Needs Theme

Staff training was the most frequently mentioned need to improve patient safety. Indeed, 17 of the 20 participants reported the importance of staff training to facilitate the patient safety climate. For example, a Saudi nurse stated: “*This will help us because it will decrease our incidents against our patients, incidents against our staff, and will give us some alternative or critical thinking for us on how we will solve problems*” (SN3).

This need was seen to be especially important when it comes to new staff as highlighted by one Saudi nurse: “[Patient safety] is not clearly discussed with our staff during their orientation program, or during the interview, usually we are focusing only on their experience, and their qualifications” (SN1).

Some participants also reported other safety needs under the subthemes of guidelines and protocols. For example, one nurse stated: “*For me, the important and safe practices in the area, or in our work is based on the protocol, policies and procedures of the hospital*” (NSN3).

## 4. Discussion

The findings from qualitative analysis showed doctors and nurses held some similar safety climate attitudes with respect to the safety needs of their department, the need for facilities and resources, and barriers to patient safety. However, nurses reported issues with doctors with respect to their teamwork, communication, and attitudes towards patients. As found in other research [5,6], nurses reported that their input was not well received, they wereunderestimated by doctors, and disagreements between physicians and nurses are not well reconciled. At the same time, the doctors often did notelaborate their answers to questions on patient safety climate; preferring to share platitudes and take a non-committal stance on many safety attitudes.

Nurses may report issues with doctor’s patient safety attitudes because doctors are less engaged with the issue and spend less time in direct patient care. This finding is perhaps not surprising given a survey of medical school programmes indicated patient safety education is lacking within many medical school programs, despite an acknowledgement of the need for such education [22]. By implication, doctors may be less engaged in safety climate issues because safety concerns are peripheral to their training. As noted by 17 of the 20 participants, it is important to conduct and participate in ongoing staff safety training to facilitate the patient safety climate in a general sense. At the same time, the literature [23] also suggests that safety training should be further incorporated into medical school curricula for doctors as part of their professional development and to improve their attitudes towards patient safety.

The finding of under-reporting medical errors is consistent with other research [24,25]. In a systematic review [25], healthcare personnel, especially doctors, were found to under-report errors for fear of individual and legal blame. Similarly, participants in this study perceived under-reporting of errors by doctors and identified several barriers to error reporting that have the potential to impact negatively on patient safety. These included attributions of blame, management viewing the reporting of errors as the reporting of complaints, a lack of accountability (especially among doctors), and inadequate follow-up on incident reporting. Whereas accurate incident reporting is an important objective in a positive hospital safety climate, the findings suggest several processes may undermine this objective and ultimately compromise patient safety. Applying strategies to remove barriers to reporting of medical errors are likely to improve patient safety climate attitudes among doctors and nurses from hospital emergency departments. As shown by the interview findings, doctors and nurses clearly saw the importance of removing the fear of blame, providing further education, and timely feedback and action from safety incident reporting (for example, Occurrence Variance Reports) as important elements contributing to a positive patient safety climate.

An important safety climate attitude to emerge from the findings, and also shown in other research [5,6,7], was the comparatively negative evaluations about management support; hospital management was perceived to be not doing enough to support safety in the emergency department. These findings suggest that interventions to improve management support and engagement would lead to more positive patient safety climate attitudes among both doctors and nurses. Indeed, research has shown that an intervention that entailed executive walk rounds to review safety hazards and ensure staff had the resources and support to implement safety interventions led to improvements in safety climate attitudes among hospital medical staff [26].

## 5. Limitations

The use of semi-structure interviews may have increased the likelihood of response inhibition in participants [27] in response to a fear of negative repercussions arising out of open and honest responding. Moreover, the setting for the study limits the generalizability of the findings to other hospital contexts. A further limitation was there were fewer substantive comments from doctors than nurses.

## 6. Conclusions

The findings of this study provide one of the few research contributions to knowledge on the patient safety climate attitudes of Saudi and non-Saudi doctors and nurses in hospital emergency departments and the factors that may impede or facilitate patient safety climate attitudes.These factors include limits to resources, teamwork, communication, and incident reporting. The findings suggest the application of such knowledge would be desirable given the importance of safety climate attitudes forenhancing positive patient outcomes in emergency departments.

## Figures and Tables

**Table 1:** Themes and subthemes developed from analysis of interview content.

Code Name	Description
**Safety evaluation**	Overall view of the patient safety climate
**Safety barriers**	Factors that impede patient safety climate
Accountability/blame	Staff with a lack of accountability and fear of reporting errors
Language	Language barriers to safety climate
Medical errors	Types of medical errors that impact on patient safety
**Safety facilitation**	Factors that facilitate safety climate
Communication	Communication between staff and patients
Management	The role of management in facilitating safety
Protocols andincident reporting	How protocols and incident reporting impact on safety climate
Teamwork	The importance of teamwork to safety
**Safety issues**	Issues that impact negatively on safety climate
Facilities/equipment/resources	Safety issues with fixed assets and equipment
Overcrowding overload	Inadequate staff-patient ratios leading to overload
Uncompliant staff	Staff issues that impact on safety climate
Safety needs	Factors that reflect needs to improve safety
Guidelines protocols	Importance of protocols for safety
Lectures and training	The importance of training to safety climate

*Notes*: The “code name” includes themes (in bold) and subthemes.
